# Advances in the Toxicity Assessment of Silver Nanoparticles Derived from a *Sphagnum fallax* Extract for Monolayers and Spheroids

**DOI:** 10.3390/biom14060611

**Published:** 2024-05-22

**Authors:** Liliya Iskuzhina, Svetlana Batasheva, Marina Kryuchkova, Artem Rozhin, Mariya Zolotykh, Rimma Mingaleeva, Farida Akhatova, Anna Stavitskaya, Kirill Cherednichenko, Elvira Rozhina

**Affiliations:** 1 Institute of Fundamental Medicine and Biology, Kazan Federal University, Kreml uramı 18, 420008 Kazan, Republic of Tatarstan, Russia; iskuzhina.l@yandex.ru (L.I.); svbatasheva@gmail.com (S.B.); maricshka80@gmail.com (M.K.); rozhinartemkzn@gmail.com (A.R.); mazolotykh@kpfu.ru (M.Z.); rimma.mingaleeva@gmail.com (R.M.); akhatovaf@gmail.com (F.A.); 2 Institute for Regenerative Medicine, Sechenov University, Trubetskaya Str. 8/2, 119992 Moscow, Russia; 3 Department of Physical and Colloid Chemistry, Gubkin Russian State University of Oil and Gas, 119991 Moscow, Russia; stavitsko@mail.ru (A.S.); kchered@mail.ru (K.C.)

**Keywords:** extract-stabilized nanoparticles, cytotoxicity, *Sphagnum fallax*, FTIR, 3D cell culture

## Abstract

The production of nanomaterials through environmentally friendly methods is a top priority in the sustainable development of nanotechnology. This paper presents data on the synthesis of silver nanoparticles using an aqueous extract of *Sphagnum fallax* moss at room temperature. The morphology, stability, and size of the nanoparticles were analyzed using various techniques, including transmission electron microscopy, Doppler laser velocimetry, and UV-vis spectroscopy. In addition, Fourier transform infrared spectroscopy was used to analyze the presence of moss metabolites on the surface of nanomaterials. The effects of different concentrations of citrate-stabilized and moss extract-stabilized silver nanoparticles on cell viability, necrosis induction, and cell impedance were compared. The internalization of silver nanoparticles into both monolayers and three-dimensional cells spheroids was evaluated using dark-field microscopy and hyperspectral imaging. An eco-friendly method for the synthesis of silver nanoparticles at room temperature is proposed, which makes it possible to obtain spherical nanoparticles of 20–30 nm in size with high bioavailability and that have potential applications in various areas of human life.

## 1. Introduction

Currently, silver nanoparticles (AgNPs) are considered one of the most promising candidates as an antibacterial agent and about 500 tons/year of nanosilver are already being produced, and this amount continues to grow steadily [1]. AgNPs are used in electronic devices, textiles, dressings, medical devices, and disinfectants [2]. However, during the production and manufacturing process of nanosilver products, they can directly enter the environment and affect humans [3]. AgNPs are thought to exhibit biocidal effects through the slow release of Ag + and also through multiple mechanisms (such as interaction with thiol groups in proteins, the inhibition of DNA replication, and the induction of oxidative stress), making it difficult for bacterial strains to develop resistance to AgNPs [4]. Therefore, research into improving the synthesis of silver nanoparticles and analyzing their cytotoxicity, including the use of three-dimensional cell models, is relevant. 

The use of various biological components for the production of silver nanoparticles has already been described, including plant extracts [5,6], fungi [7,8], and bacteria [9,10]. Plants are a well-known source of antimicrobial and antioxidant agents [11]. However, there are few studies on moss plants. It is known that peat from *Sphagnum* sediments contains polysaccharides and highly reactive carbonyl groups. These substances help to retain nutrients and acidify the environment, which inhibits the growth of microorganisms and vascular plants [12]. In addition, studies on the composition of *Sphagnum* secondary metabolites have also been conducted [13,14]. The use of *S. fallax* is advantageous because this moss contains compounds with biological activity. The analysis of the 1H NMR spectra of *Sphagnum* metabolites showed that they include the following compounds: fructose, glucose, malate, glycerin, methylamine, propylene glycol, O-glycoside luteolin, the isomer kaempferol, as well as caffeylquinic acid. Most of these compounds possess biological activity, according to the KEGG database (https://www.genome.jp, accessed on 20 March 2024). Screening against a database of antimicrobial compounds showed the similarity of chemical compounds between Sphagnum and medicinal plants [15]. The antimicrobial properties of Sphagnum moss were similar to those of chia, followed by chamomile and hemp extracts. It has been shown that some compounds from aqueous extracts of Sphagnum mosses have a high antioxidant activity, which may result in the beneficial properties of the nanoparticles produced using *S. fallax*.

The optimization of the nanomaterial production process to reduce its negative impact on the environment is of high priority in the modern development of nanotechnology. Chemical and physical methods for nanomaterial synthesis are characterized by high energy consumption and often involve the use of ecotoxic reagents and a large number of auxiliary chemicals [16,17,18]. As a result, there is a growing demand for the use of sustainable and safe nanoparticle production methods, especially for making silver nanoparticles [19,20,21]. In a previous study, metallic silver and iron nanoparticles were prepared using an eco-friendly aqueous extract of moss (*S. fallax*), without the use of toxic reducing agents [22]. However, during the synthesis process, the solutions were heated to 90 °C for 2 h, which is not in accordance with the basic principles of green chemistry [23].

In this study, the optimization of a methodology for the synthesis of silver nanoparticles using a *S. fallax* moss extract at room temperature is presented. The metabolites on the surface of the nanoparticles are analyzed using FT-IR spectroscopy and qualitative reactions. Lung carcinoma cells (A549) and immortalized mesenchymal stem cells are used as model cultures to evaluate the toxicity of the extract-stabilized and citrate-stabilized nanomaterials. The cytotoxicity of the nanoparticles produced using different reducing agents is compared at short and long exposures. The cell morphology is evaluated, and the cell viability is studied using a set of colorimetric tests and flow cytometry. Additionally, the uptake of nanoparticles by monolayer cells is analyzed using scanning electron microscopy and dark-field microscopy. The effect of cell incubation with citrate-stabilized and extract-stabilized silver nanoparticles on the formation of multicellular spheroids is also assessed.

## 2. Material and Methods

### 2.1. Chemicals

The chemicals used in this study were purchased from Sigma-Aldrich, St. Louis, MI, USA, unless otherwise stated. All reagents were used as received, without additional purification.

### 2.2. Cell Culture

The study used epithelial-like human lung carcinoma cells (A549) (ATCC, Manassas, VA, USA) and a genetically stable immortalized MSC line derived from adipose tissue (iMSC). A combination of hTERT overexpression with TP53 knockdown was used to modify the cells [24]. The cells were cultured in DMEM medium with the addition of 10% inactivated calf embryonic serum (HyClon, Logan, UT, USA), 45 U/mL penicillin, and 45 mg/mL streptomycin (Paneco, Moscow, Russia) under the conditions of 5% CO_2_ at 37 °C in a CO_2_ incubator.

### 2.3. Obtaining the Sphagnum fallax Moss Extract

*Sphagnum fallax* (H. Klinggr.) moss was collected from a natural source—an oligotrophic swamp in the Ulyanovsk region (Maloe Swamp). In the laboratory, the plants were rinsed with running and distilled water and dried. The dried moss *S. fallax* (1.3 g) was placed in a conical flask with a capacity of 100 mL (borosilicate glass Rasotherm, Jenaer Glas, GDR) together with 55 mL of distilled water, previously brought to a boil on an electric stove. The contents of the flask were boiled for 5 min; then, the plants were extracted from the liquid in sterile laminar conditions (laminar flow cabinet of the 2nd class of protection SafeFAST Elite 215 S, Faster, Ferrara, Italy) and squeezed into the flask using laboratory instruments. The resulting extract was allowed to settle and cool in sterile conditions for 30 min. After that, the supernatant was transferred to a 15 mL tubes (EasyFlip Centrifuge tubes, JET Bio-Filtration Products Co, Guangzhou, China) using a serological pipette. The tubes were centrifuged (CM-6MT, ELMI, Riga, Latvia) for 5 min at 700 g and the sediment was discarded. The supernatant (the moss extract) was sealed using a laboratory film (Parafilm M, Bemis Company, Sheboygan Falls, WI, USA) and stored in the dark at 4–5 °C.

#### 2.3.1. Extraction Method

Extract-stabilized silver nanoparticles were synthesized by reducing silver ions using the sphagnum extract, which acted as a reducing agent and a stabilizer. The dry AgNO_3_ powder was dissolved in distilled water at a concentration of 1 mmol/L. The solution was then sterilized in an autoclave sterilizer (Prestige Medical Ltd., Blackburn, UK). For the biosynthesis of silver nanoparticles stabilized with a plant component, an aqueous moss extract and a 1 mM aqueous solution of AgNO_3_ were used. These were mixed under sterile conditions at a ratio of 1:1, 1:2, and 1:4 and kept at room temperature in the dark. The moss extract and 1 mM AgNO_3_ aqueous solution in the ratio of 1:2 turned out to be the most effective for the formation of nanoparticles, and this ratio was used for our subsequent work. The formation of silver nanoparticles was monitored visually by observing changes in the color of the suspension. The color of the suspension changed from pale yellow to dark brown (Figure S1, Supplementary Materials). The nanoparticles were then washed several times with distilled water (20 min, 17,000× *g*) and dried in a sterile environment. Only the washed nanoparticles were used in all the experiments.

#### 2.3.2. Citrate Method

An aqueous solution of AgNO_3_ (0.012 M, 100 mL) was heated to 90 °C with a magnetic stirrer. While stirring, 10 mL of 5% sodium citrate was added dropwise. After adding all the sodium citrate, the resulting mixture was continuously stirred at 90 °C for 2 h. After 10 min of the reaction, the mixture color changed from yellow to dark gray, indicating the formation of silver nanoparticles. The resulting solution was left for 12 h at room temperature until completely cooled, and then for 24 h to mature. Citrate salts were removed by dialysis. The particles were separated by centrifugation at 17,000× *g* for 60 min.

### 2.4. Characteristics of AgNPs

#### 2.4.1. Optical Absorption Spectroscopy

The optical properties of the nanoparticles were investigated using UV-vis absorption spectroscopy (NanoPhotometer NP80 Touch, Implen, München, Germany), using 10 mm quartz cuvettes.

#### 2.4.2. Nanoparticle Size

Microimages of nanoparticles were obtained using the Dimension Icon atomic force microscope (Bruker, Billerica, MA, USA) in the PeakForce Tapping mode at room temperature. Standard silicon nitride ScanAsyst-Air (Bruker) cantilevers with optimal scanning parameters, such as scanning force of 1–2 nN at a scanning rate of 0.8–0.9 Hz, were used. Image processing was performed using the Nanoscope Analysis v 1.7 software (Bruker).

Additionally, the morphology of the nanoparticles was studied using a JEM-2100 electron microscope (JEOL, Tokyo, Japan) operating in the transmission electron microscopy (TEM) mode with a magnification factor of 50–1,500,000 times and an image resolution of 0.19 nm at a voltage of 200 kV. The sample holder was cleaned in a plasma chamber to prevent organic compounds from entering the microscope. To determine the average particle size, micrographs were processed using the Image J software V 1.8.0.

Scanning transmission electron microscopy (STEM) in combination with energy dispersive X-ray spectroscopy (EDX) was performed on the JEM-2100 instrument (JEOL, Tokyo, Japan), operating in the STEM mode.

#### 2.4.3. Stability of the Nanoparticles

The stability of the silver nanoparticle suspensions was assessed by measuring the sedimentation rate, which was determined by changes in the light transmission coefficient over a period of 7 days at a AgNP concentration of 0.25 mg/mL. 

The required pH value (3.0–9.0) of the solutions was achieved by adding either hydrochloric acid or sodium hydroxide solutions (0.1 mol/L) and controlling the pH with a SevenCompact S220 pH meter (Mettler Toledo, Im Langacher, Greifensee, Switzerland). The stabilities of the moss extract- and citrate-stabilized AgNPs were determined at room temperature; the particles were suspended in ultrapure water obtained using the Simplicity^®^ Water Purification System (Merck, Darmstadt, Germany).

The hydrodynamic diameters and zeta potentials of the nanomaterials used were determined using a Zetasizer Nano ZS device (Malvern Panalytical, Malvern, Great Britain) at a pH of 5.5 and temperature of 25 °C.

#### 2.4.4. Dark-Field Microscopy with Hyperspectral Analysis

Dark-field images and reflected light spectra of silver nanoparticles were obtained using an Olympus BX51 (Olympus, Tokyo, Japan) microscope equipped with an enhanced CytoViva^®^ dark-field condenser and a 150 W halogen Fiber-Lite DC-950 (Dolan-Jener, Boxborough, MA, USA) light source. 

The dark-field images were obtained at an exposure time of 154 ms and 575 ms using Exponent 7 software (Dage-MTI, Michigan City, IN, USA). The spectra were recorded using a Specim V10E spectrograph (Spectral Imaging Ltd., Oulu, Finland) and a Pixelfy.usb CCD camera in the wavelength range from 400 to 1000 nm with a spectral resolution of 2 nm. The HSI data were collected from 700 scan lines at 0.25 s exposure and full illumination intensity, which took about 9 min to obtain a full scan. The hyperspectral data (HSI) were stored and analyzed as hypercubes using the ENVI v. 4.8 software (Harris Geospatial Solutions, Broomfield, CO, USA) [15]. The HSI data were processed using the software internal correction algorithm to eliminate the lamp spectra. The averaged spectra were obtained from 50 individual particles of all colors (1000 pixels) and were used to create a spectral library. The spectral library obtained was used for the SAM classification of the nanoparticles in the cells and spheroids.

Dark-field hyperspectral microscopy was also performed to assess the distribution of the silver particles in the iMSC and A549 cell lines. The mapping of the particles was carried out using the ENVI v. 4.8 software Spectral Angle Mapper (SAM) algorithm [25]. The SAM algorithm was used in the Single Value mode, with the maximum angle set to 1 radian.

#### 2.4.5. Fourier Transform Infrared Spectroscopy

The IR spectra were registered using the FT-801 Fourier-IR spectrometer equipped with an attenuated total reflection (ATR) unit with ZnSe crystal (Simex, Novosibirsk, Russia) and processed with the ZaIR 3.5 software (Simex, Novosibirsk, Russia). Each spectrum was averaged from 26 scans registered at a resolution of 4 cm^−1^. The sample was applied to the ATR stage in the form of a solution or suspension, and then dried in air.

### 2.5. Phytochemical Analysis

The analysis of organic compounds in *Sphagnum fallax* moss extract was carried out using standard methods [26,27]. 

#### 2.5.1. Test for Phenolics

To determine phenols, several drops of a 5% solution of FeCl_3_ × 6H_2_O were added to the moss extract. The presence of phenolic compounds was indicated by a change in the solution color to a blue-black color.

#### 2.5.2. Test for Terpenoids

To determine terpenoids, chloroform and then H_2_SO_4_ were added to the plant extract. The formation of a reddish-brown color at the interface of both chemicals indicated the presence of terpenoids.

#### 2.5.3. Test for Glycosides

To detect glycosides, 1 mL of plant extract was diluted with 1 mL of water and then 0.5 mL of lead acetate solution was added. Then, the resulting mixture was filtered, and CHCl_3_ was added and left for 15–20 min. After the evaporation of CHCl_3_, iron chloride, glacial acetic acid, and concentrated sulfuric acid were added to the test solution. The appearance of two separate layers in the test tube—the lower reddish-brown layer, which changed to bluish-green, and the upper layer of acetic acid, which remained the same—indicated the presence of glycosides.

#### 2.5.4. Test for Saponins

To detect saponins, 0.5 mL of the moss extract was diluted with distilled water to obtain a total volume of 10 mL. The mixture was then vigorously shaken for at least 2 min. The presence of saponins was confirmed by the appearance of creamy small bubbles, such as foam.

#### 2.5.5. Detection of Tannins

To detect tannins, 1 mL of the moss extract and 1 mL of water were diluted and 2–3 drops of a 10% solution of ferric chloride were added to it. The formation of brownish-green and bluish-black sediments indicated the presence of tannins.

#### 2.5.6. Test for Alkaloids

To determine the alkaloids, concentrated hydrochloric acid (4–5 drops) was added to 1 mL of the moss extract. After the thorough mixing of the reaction mixture, it was treated with Dragendorff reagent, which had previously been prepared from bismuth nitrate, acetic acid, and potassium iodide according to a standard protocol [28]. The appearance of an orange color indicated the presence of alkaloids.

### 2.6. Assessment of Cytotoxicity

#### 2.6.1. Cell Viability Assessment (MTT Analysis)

The MTT (4-5-dimethylthiazolyl-2)-2,5-diphenyltetrazolium bromide assay was used to assess the cell viability. The cells were seeded in 96-well plates (3000 cells per well) and incubated at 37 °C for 24 h with 5% CO_2_. After that, a cytometer (Tali Image-Based Cytometer, Thermo Fisher Scientific, Waltham, MA, USA) was used to count the living cells. The MTT method is based on the ability of the enzyme succinate dehydrogenase of mammalian mitochondria to reduce MTT to purple formazan crystals in the cytoplasm of living cells. After the incubation of cells with silver nanoparticles at different concentrations for 24 h, the MTT solution was added to the wells and incubated for 4 h. After the removal of the medium, the resulting insoluble formazan was extracted and the optical density was estimated at a wavelength of 540 nm on a multifunctional photometer (Multiskan FC, Thermo Fisher Scientific, Waltham, MA, USA).

#### 2.6.2. Cell Viability Analysis with the Resazurin Reduction Assay

This method is based on the ability of living cells to reduce the blue low-fluorescent resazurin. During the reduction by living cells, rezazurin is converted into the fluorescent derivative resorufin. The intensity of resorufin fluorescence at a wavelength of 570 nm is directly proportional to the number of cells and their respiratory activity. A549 and iMSC cells were seeded with nanoparticles into 96-well plates (7000 cells per well) and cultured in a humid atmosphere with 5% CO_2_ at 37 °C for 24 h. Cells seeded without nanoparticles were used as the controls. The culture medium was removed and replaced with 200 µL of resazurin (7-hydroxy-3H-phenoxazine-3-oh 10-oxide) solution (Sigma-Aldrich) in the culture medium, and the plates were incubated overnight. Absorption was measured at 570 nm using a microplate reader. The rate of resazurin reduction is directly proportional to the number of viable cells.

#### 2.6.3. Visualization of the Cells

Cell morphology and monolayer formation were observed during the cultivation of nanoparticle-treated and intact cells for 24 h using dark-field and scanning electron microscopy.

The interaction of the cells with the nanoparticles was recorded using scanning electron microscopy (Carl Zeiss, Oberkochen, Germany). The cells were dehydrated using a series of alcohols, and the samples were sprayed with an alloy of Au (60%) and Pd (40%) using a Q150R device (Quorum Technologies, Puslinch, ON, Canada). The images were obtained at an operating pressure of 3 × 10^−4^ Pa and an accelerating voltage of 15 kV using the InLens detection mode.

The effect of the silver nanoparticles on the integrity of cell membranes was evaluated using the trypan blue exclusion method. The cells were incubated in a 96-well culture plate in the presence of nanoparticle synthesis precursors and various concentrations of silver nanoparticles (1, 6 and 12 µg/mL) in three replications for 24 h. After that, the cells were incubated for 2 min with 4% trypan blue in a culture medium. It is assumed that the dye only stains cells with a damaged cell membrane. Using an inverted light microscope, the percentage of destroyed (dead) and intact (viable) cells was estimated. When using the trypan blue exclusion method to assess the cell viability, a field with a large number of cells is always visualized. When looking at large fields of view, one can obtain a more representative impression of the specimen because a larger number of cells or structures is observed. This helps to avoid artifacts caused by random anomalies in individual areas. Additionally, small areas at high magnification include only a part of cell population and do not always represent the real distribution of differently colored cells in the specimen. Thus, analyzing large fields of view provides a more objective and reliable assessment of silver nanoparticle cytotoxicity.

#### 2.6.4. Assessment of the Cell Index

The impedance of the cells was measured using the xCELLigence real-time cell analyzer (Roche, Mannheim, Germany). The cells were grown for 24 h in special culture E-cups (E-plate, Sigma, Burlington, MA, USA), and the cell index was measured every hour. The cells were seeded with 5000 cells per well, and after 24 h, AgNPs at concentrations of 1, 6, and 12 µg/mL were added.

#### 2.6.5. Induction of Necrosis

The viability of the cells after exposure to silver nanoparticles was studied by flow cytometry using the BD FACS device (Franklin Lakes, NJ, USA). Propidium iodide (PI) dye (Invitrogen, Carlsbad, CA, USA) was used for the contrast staining of dead cells. Propidium iodide is known to stain late apoptotic and necrotic cells that have lost the membrane integrity [29]. The cells were seeded into 6-well plates (1 × 10^5^ cells per well) and incubated for 24 h. Silver nanoparticles were added to the cells at various concentrations, and the cells were additionally incubated for 24 h. The cells were then collected, washed twice with PBS, treated with PI (propidium iodide), and analyzed.

### 2.7. Spheroids

The hanging drop method was used to form spheroids [30]. Briefly, 1000 cells were used to form multicellular spheroids. The cells were mixed with nanoparticles and cultured in hanging drops for 7 days in a humid atmosphere with 5% CO_2_ at a temperature of 37 °C, as described in our previous study [31]. The distribution of silver nanoparticles within spheroids was visualized on day 7 of the experiment.

### 2.8. Statistical Analysis

All data were expressed as averages ± SD. GraphPad Prism version 9 was used for statistical treatment. The differences between the groups were shown as ns (insignificant), * *p* < 0.05, ** *p* < 0.01, and *** *p* < 0.001.

## 3. Results and Discussion

**Figure 1:**
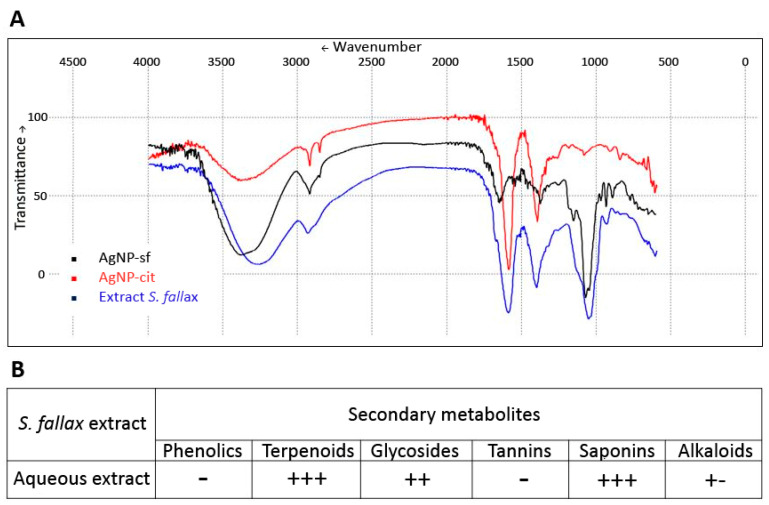
FTIR spectra of the *S. fallax* extract and citrate-stabilized (AgNP-cit) and extract-stabilized (AgNP-sf) silver nanoparticles (**A**); phytochemical composition of *S. fallax* moss extract (**B**). The symbols “+” and “-” denote the degree of reaction intensity.

The surface properties of silver nanoparticles obtained with citrate or the sphagnum extract were evaluated using FT-IR spectroscopy. This technique is often used to identify the functional groups that are present in active plant ingredients [32]. In the IR spectrum of the sphagnum extract (Figure 1A), the wide and intense peak in the region of 3500–2500 cm^−1^ indicates the presence of hydroxyl groups, and the very intense band at 1051 cm^−1^ most likely belongs to C-O stretching vibrations in alcohol or ether bonds. A peak in the range of 650 ± 50 cm^−1^, corresponding to the out-of-plane O-H bending vibrations, also indicates the presence of alcohol. However, all the above bands are rather wide and look like envelopes over several bands, corresponding to many C-O and O-H bonds with slightly different characteristics, like is often observed for polysaccharides. The two intense bands of about 1587 and 1397 cm^−1^ correspond to asymmetric and symmetrical carbon–oxygen stretching vibrations in carboxylates, respectively. There are also two small bands at 1521 and 1507 cm^−1^ and a little shoulder at 1541 cm^−1^, which could imply that there can be bands for secondary amide bonds hidden by the large carbon–oxygen band at 1587 cm^−1^. The blob at 2929 cm^−1^ results from C-H stretches in saturated carbon compounds. Thus, the IR spectrum of the sphagnum extract indicates the presence of carboxylates and possibly oligo- or polysaccharides, peptides, and/or proteins. 

In the spectrum of the AgNPs stabilized with citrate, the wide peak at about 3500 cm^−1^ indicates the presence of OH- groups, and there is also the O-H out-of-plane band at 660 cm^−1^. The two bands at 2917 and 2849 cm^−1^ correspond to asymmetric and symmetric C-H stretching vibrations in the methylene group, respectively. The intense bands at around 1584 and 1393 cm^−1^ probably belong to –COO– groups in carboxylates, whereas the low intensity peaks below 1400 cm^−1^ include C-C stretching bands. This band pattern is in a good agreement with the presence of the citrate residues that were used for stabilizing AgNPs.

In the spectrum of the AgNPs stabilized by the extract, there is a wide and intense band around 3400 cm^−1^ corresponding to O-H stretching vibrations. The two bands at 2917 and 2850 cm^−1^ belong to C-H stretching vibrations in the methylene group. The peaks between 1200 and 1000 cm^−1^, including the two dominant peaks at 1069 and 1046 cm^−1^, probably correspond to C-O asymmetric stretches in alcohols, ethers, or carbohydrates, which contain both ether bonds and alcohol groups. The band at 886 cm^−1^ is probably the C-O symmetric stretch. The two intense peaks at 1647 and 1373 cm^−1^ most probably correspond to carbon–oxygen bonds. There is also a rather intense band at 1541 cm^−1^. The bands at 1647 and 1541 cm^−1^ are usually encountered in the spectra of secondary amide groups. The first peak belongs to a C=O stretch, while the second one is an in-plane N-H bend. Thus, the bands at 1647 and 1541 cm^−1^ indicate the presence of peptides or proteins on the nanoparticles and also support our earlier assumption that there were peaks corresponding to amide bonds hidden in the spectra of the sphagnum extract. The similarity of the spectra of the sphagnum extract and the AgNPs synthesized in the presence of the sphagnum extract confirms the modification of the AgNPs with the extract components. As the particles were washed thoroughly with dH_2_O before the FTIR analysis, all the above data refer to substances adhered to the nanoparticles. The FTIR analysis demonstrated that the main compounds that adhered to the nanoparticles were most probably peptides or proteins and oligo- or polysaccarides. Thus, the extract not only served as a reducing agent but also provided substances that modified the nanoparticles.

The analysis of the phytochemical composition of the *S. fallax* moss extract allowed to identify key components that could influence the synthesis of the AgNPs (Figure 1B). It was found that the extract lacks phenolic and tannic compounds, but at the same time contains a significant amount of terpenoids, saponins, and glycosides, as well as a small amount of alkaloids. Thus, the *Sphagnum* moss extract was found to be rich in phytochemical compounds, which is consistent with the results of previous studies [33].

**Figure 2:**
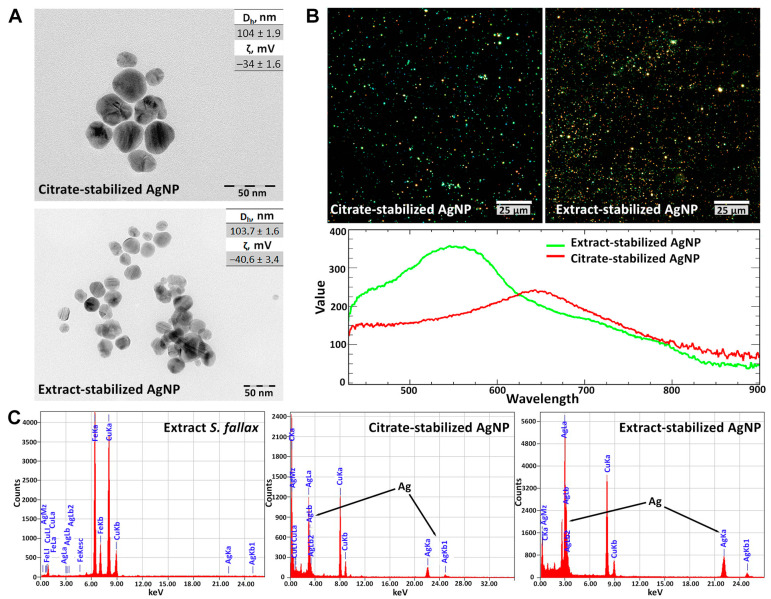
Characterization of silver nanoparticles (AgNPs). Microimages of the AgNPs obtained by transmission electron microscopy and the values of hydrodynamic diameter (Dh) and zeta potential (ζ) of the AgNPs (**A**). Dark-field microimages and reflection spectra of the AgNPs (**B**). EDX analysis of the silver nanoparticles and the *S. fallax* moss extract (**C**).

It is known that the most important characteristics of nanomaterials are their size and shape, which determine the colloidal stability of nanomaterial suspensions [34,35]. The properties of nanoparticles characterized by different methods are summarized in Figure 2. The size and morphology of the synthesized AgNPs were observed using TEM (Figure 2A). It showed that the AgNPs had a spherical shape and were well dispersed. The measurements of the nanoparticle size in the TEM images showed that the nanoparticles obtained using both types of reducing agents were about 20–30 nm in size. In addition, the hydrodynamic diameter (Dh) and zeta potential (ζ) were determined by dynamic light scattering (DLS) and Doppler laser velocimetry. It is interesting to note that plasmonic nanoparticles exhibit size-dependent spectral characteristics that cannot be detected using bright field microscopy but can be studied using dark-field microscopy (Figure 2B). The hyperspectral analysis allows the identification of isolated or aggregated nanoparticles in aqueous solutions [36] and inside living cells [37]. The presence of silver nanoparticles in both samples was confirmed by STEM-EDX (Figure 2C), which demonstrated strong signals in the range of 2.5–4 and 20–25 keV, corresponding to major emission energies for silver [38].

The formation of silver nanoparticles was additionally confirmed by UV–visible spectroscopy in the wavelength range of 200–900 nm. It was shown that the citrate-stabilized and extract-stabilized samples had absorbance peaks λmax at 405–420 nm and a reddish-brown color, indicating the presence of AgNPs (Figure S2, Supplementary Materials). The absorption peaks of the AgNPs are associated with the surface plasmon resonance phenomenon. The plasmon resonance is an intrinsic property of AgNPs and results from the interaction of the electron cloud on the AgNP surface with incident electromagnetic radiation and typically occurs in the range from 380 to 420 nm, depending on the size of the AgNP analyzed [39]. 

The stability of the obtained AgNPs was evaluated at different pH values at room temperature. The hydrodynamic diameters and zeta potentials determined (Figure S2, Supplementary Materials) at different pH values showed the high stability of the obtained nanoparticles.

### 3.1. Effects of Extract-Stabilized and Citrate-Stabilized AgNPs on the Physiological Properties of Human Cells

The literature describes the production of silver nanoparticles with various sizes (ranging from 5 to 100 nm) and morphologies (spherical, nanorods, hexagons, etc.) using different plant components, such as soluble starch, coffee, green tea, mushrooms, spices, and other biological extracts [40,41,42,43,44,45]. Moreover, it has been suggested that biogenic AgNPs may be less toxic in vivo than AgNPs synthesized using pure chemicals [46]. It should be noted that chemical reduction of silver ions is still the most widely used method for the production of nanoparticles. This simple technique provides reliable control over the experimental parameters, resulting in the formation of nanoparticles with small sizes and low polydispersity [47,48]. This highlights the need for a thorough comparative analysis of nanoparticles synthesized using different reducing agents. At the same time, there is a concern about the increasing use of silver-based nanomaterials due to their potential negative effects on the environment and human health, while the mechanisms of exposure to AgNPs are still not fully understood. Therefore, it is essential to conduct extensive studies on AgNP toxicity to living organisms and their behavior and fate in the environment [49].

In this study, the effect of two different synthesis precursors, citrate and *Sphagnum* extract, as well as the nanoparticles produced using these precursors, on two types of cells, mesenchymal stem cells (iMSC) and human lung adenocarcinoma cells (A549), was analyzed in 24 h after their introduction into the cell incubation medium. The choice of cell lines is justified by their relevance for studying the effects of nanoparticles on both cancer and normal cells. These cells are well-studied and have a high rate of cell division, making them ideal for this type of research [50,51]. Moreover, AgNPs are widely used in consumer aerosol products due to their antibacterial ability; so, there is a risk of their ingestion and deposition in the respiratory tract [32]. Initially, standard colorimetric assays, the MTT test and the rezazurin reduction method, were used to assess the cytotoxic effect of silver nanoparticles on mammalian cells. It is known that MTT analysis is based on the conversion of the water-soluble yellow dye 3-(4,5-dimethylthiazole-2-yl)-2,5-diphenyltetrazolium bromide (MTT), which, when reduced by dehydrogenase and reducing agents present in metabolically active cells, results in water-insoluble purple formazan, which can be extracted with organic solvents and evaluated using spectrophotometry [52,53,54]. Unlike MTT, rezazurin is reduced by a wider range of enzymes; in addition to mitochondrial dehydrogenases, it can also be reduced by cytochromes and dehydrogenases localized in the cell cytoplasm [55]. The combination of these methods allows for a more accurate assessment of the effect of different types of nanoparticles on cell metabolism. 

In both MTT and resazurin assays, the influence of AgNPs on the light absorption by the dyes was also tested in the specimens that contained only the cell medium and AgNPs and did not contain cells. No changes in the light absorption compared to the absorption of the dyes in the pure cell medium (without AgNPs or cells) were found. This result is in an agreement with those of other studies, where silver nanoparticles influenced the results of the MTT assay only at concentrations higher than 50 mg/mL [56].

**Figure 3:**
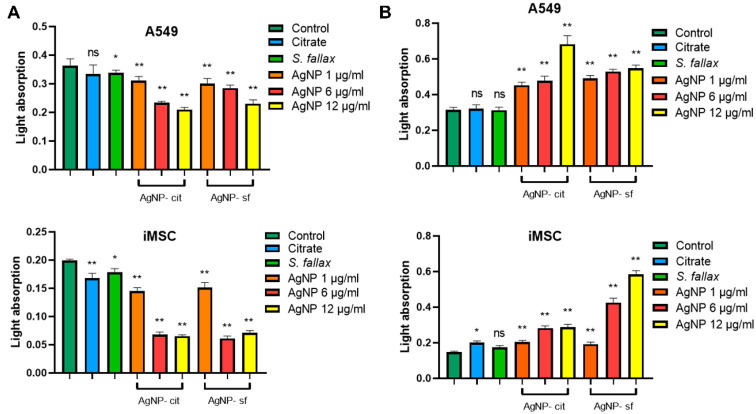
Changes in the physiological state of A549 and iMSC cells after 24 h of incubation with sodium citrate, *S. fallax* moss extract, and citrate-stabilized (AgNP-cit) and extract-stabilized (AgNP-sf) silver nanoparticles at concentrations of 1, 6, and 12 µg/mL. MTT test (**A**); resazurin reduction assay (**B**). ns: insignificant, * *p* < 0.05, ** *p* < 0.01.

The effect of the studied compounds on cells at concentrations ranging from 1 to 12 µg/mL was determined. It was shown that extract- and citrate-stabilized silver nanoparticles had a weak cytotoxicity for the studied cell types at the concentration of 1 µg/mL in both tests (Figure 3). The introduction of 6 and 12 μg/mL of silver nanoparticles, obtained by the citrate method, into the incubation medium significantly reduced the viability of A549 cells compared to those stabilized with the extract. The resazurin reduction assay also showed a similar effect of the AgNPs on the cells. It should be noted that the physiological activity of mesenchymal stem cells also decreased significantly during incubation with silver nanoparticles (6 and 12 µg/mL), while the effect did not depend on the type of reducing agent used. Different studies have shown that the effect of nanoparticles on cells varies depending on the type of the reducing agent, the nanoparticle size, and the cell type used. For example, the half lethal concentration of silver nanoparticles synthesized using tea extracts for the human colon cancer cell line HT-29 was 71.5 µg/mL, whereas for the human breast cancer cell line MCF-7, it was 59.2 µg/mL, and for normal human lung fibroblast cells (IMR-90), the LC50 of starch-coated silver nanoparticles was 50 µg/mL [57]. The authors noted the significant effect of the size of nanoparticles on the toxicity to cells. Thus, in the work of Li et al., it was shown that silver nanoparticles with a size of 60–70 nm have insignificant cytotoxicity even at a relatively high dose of 125 µg/mL. At the same time, silver nanoparticles with sizes from 25 to 45 nm in the same concentration were toxic, leading to the death of more than 50% of cells [58]. 

The differences in the results of the colorimetric tests may be due to the different activity of the metabolic pathways in A549 and iMSC cells, as well as the peculiarities of the tests performed. The data obtained showed that the cytosolic enzymes of A549 cells were suppressed to a greater extent when citrate-stabilized silver nanoparticles were used, while silver nanoparticles produced with citrate and the extract had the same cytotoxic effect on iMSC cells. When silver nanoparticles were obtained using heating, the extract-stabilized nanoparticles at a concentration of 5 μg/mL caused the death of almost 70% of iMSC cells [5].

**Figure 4:**
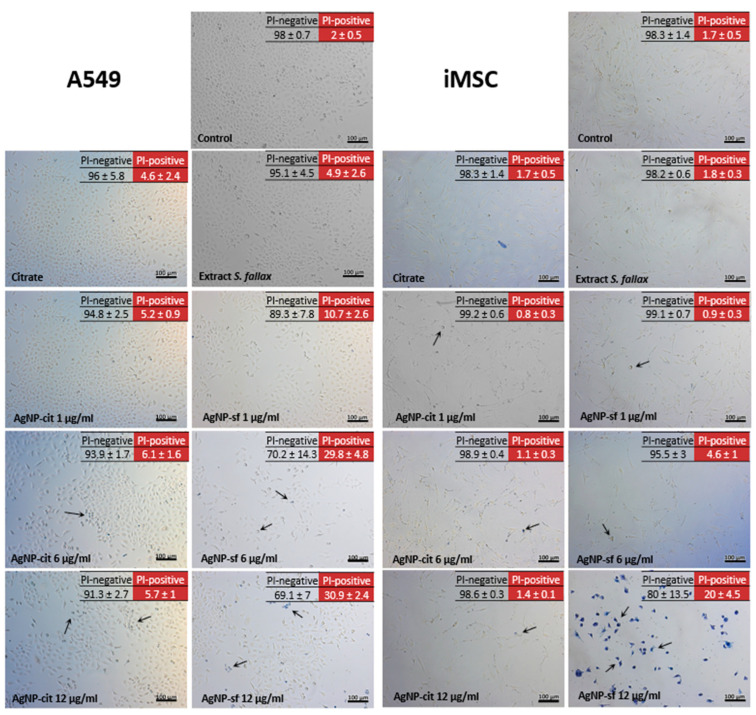
Microimages of A549 and iMSC cells treated with trypan blue and the results of the analysis with propidium iodide (in the form of a table located in the upper right corner) to determine the necrotic effect. The cells were incubated for 24 h with sodium citrate, *S. fallax* moss extract, and citrate-stabilized (AgNP-cit) and extract-stabilized (AgNP-sf) silver nanoparticles at concentrations of 1, 6, and 12 µg/mL. Arrows denote altered cells.

Next, the effects of silver nanoparticles were assessed using trypan blue staining, and the number of necrotic cells in the population was determined using flow cytometry (Figure 4). Staining with trypan blue revealed a dose-dependent cytotoxic effect of AgNPs stabilized with citrate and the *Sphagnum* extract (viable cells are colorless, and non-viable cells are blue) (Figure 4). It is noteworthy that a greater number of dead cells stained with trypan blue were observed during the incubation of both cell types with the silver nanoparticles stabilized with the sphagnum extract. In addition, the cell monolayers treated with the extract-stabilized silver nanoparticles demonstrated less surface coverage. Visual changes in the cells were observed, more corresponding to necrosis (swelling of the cytoplasm, membrane rupture, etc.).

Interestingly, citrate had a greater effect on A549 cells, significantly reducing their growth, while the effect of the *Sphagnum* extract was greater for iMSC cells. Both types of nanoparticles at a concentration of 1 µg/mL had no effect on cells. An increase in the concentration of silver nanoparticles to 6 µg/mL led to a noticeable decrease in the number of cells (Table 1) and the appearance of signs of necrosis, such as cell swelling, the destruction of the plasma membrane, and cell lysis. These changes were more pronounced in cells incubated with the extract-stabilized nanoparticles. At the maximum concentration of silver nanoparticles of 12 µg/mL, changes in the characteristic structure, loss of the total number of cells, and an increase in the number of non-viable (blue) cells become maximal. In this case, the most pronounced cytotoxicity of the extract-stabilized nanoparticles was observed. 

The table shows the results of the analysis of the surface coverage area with A549 and iMSC cells (Table 1).

As in the case of the colorimetric tests, iMSC cells were found to be more sensitive to silver nanoparticles compared to A549 cells. The necrosis induction in A549 and iMSC cell lines was evaluated using propidium iodide, which stains late apoptotic and necrotic cells that have lost membrane integrity. Flow cytometry is a method that mitigates interference associated with the unique optical, magnetic, and absorption properties of silver nanoparticles. Here, the analysis is based on the cumulative output of individually analyzed cells, while in plate-based spectrophotometric methods, the analysis readout is the result of thousands of cells in one cell culture well [59]. The results of cytometry correspond to the results of other assays, except for the trypan blue staining. Thus, it was shown that the incubation of A549 and iMSC cells with silver nanoparticles (both citrate- and extract-stabilized) led to impaired growth and caused significant morphological changes in both cell types in the form of rounding, loss of turgor, formation of uncharacteristic fibers, and ruptures. At the same time, the extract-stabilized silver nanoparticles had a more negative effect.

**Figure 5:**
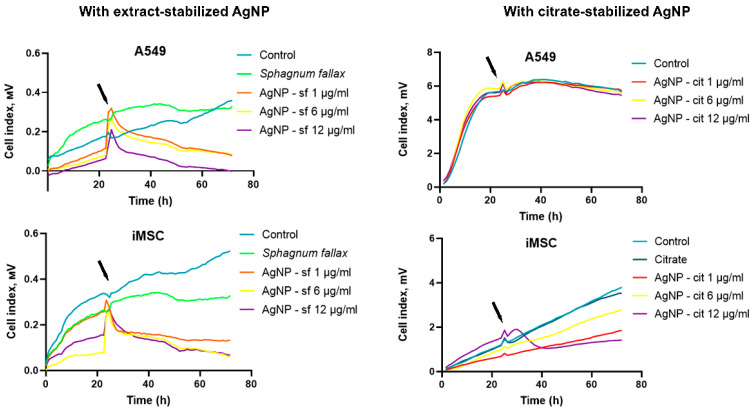
Real-time monitoring for 80 h of the cell index of A549 and iMSC cells incubated with sodium citrate, *S. fallax* moss extract, and citrate-stabilized (AgNP-cit) and extract-stabilized (AgNP-sf) silver nanoparticles at concentrations of 1, 6, and 12 µg/mL. The time of adding nanomaterials is indicated by an arrow (24 h).

It was shown that the introduction of silver nanoparticles into the incubation medium caused membrane depolarization after 60 s due to changes in the functions of calcium channels and Na+ influx channels [60]. At the same time, a number of studies report the induction of acute lung toxicity after short-term exposure to silver nanoparticles [33,61,62]. However, in view of human risk, the assessment of long-term exposure to silver nanoparticles is the most significant. Therefore, the dependence of the cellular index on the time of cell incubation was further assessed. The analysis of cell viability for 80 h demonstrated a decrease in the proliferative activity of A549 and iMSC cells in the presence of silver nanoparticles compared to the control group (Figure 5). Both cell lines were most susceptible to extract-stabilized nanoparticles. Interestingly, when exposed for a longer period of time, the toxicity of citrate-stabilized nanoparticles on A549 cells was found to be less severe than after 24 h of co-incubation. However, mesenchymal stem cells, in contrast, showed a greater sensitivity to the citrate-stabilized nanoparticles during prolonged incubation. It is known that the cell membrane contains a significant number of negatively charged sites, and the silver nanoparticles stabilized with citrate had a lower zeta potential and were more prone to aggregation. The aggregation of AgNPs is an important parameter affecting their bioavailability and cytotoxicity [63]. It is possible that the increased toxicity of extract-stabilized silver nanoparticles is associated with their stronger interaction with cell membranes. This could be an advantage for their potential future use as antibacterial agents.

In most cases, in vitro cell models, such as the A549 lung epithelial cell line and primary cells, are used to explain the pulmonary toxicity of AgNPs [64]. It is assumed that the toxicity of AgNPs is largely due to the release of Ag+ [65,66], when cations enter cells and damage the membrane, lysosomes, mitochondrial respiratory chains, or DNA [67,68]. In addition, the released silver cations can affect ion exchange in the cell membrane, block some ion pathways, and finally cause cell death [69]. The production of reactive oxygen species (ROS) is another mechanism for the achievement of AgNP toxicity, which subsequently causes cell death through apoptosis and the stimulation of autophagy. Moreover, more and more studies report that autophagy, a natural process of the degradation of cytoplasmic components, is involved in the cytotoxicity of AgNPs [37]. AgNPs can impact the autophagic flux [70,71], which can lead to increased cytotoxic reactions, including autophagic cell death and/or apoptosis. Despite the fact that there are many assumptions about the mechanism of toxic action of AgNPs, a comprehensive understanding is still lacking. In addition, many of the data obtained are contradictory, for example, Fehaid and co-authors reported on the antiapoptotic effect of silver nanoparticles on lung epithelial cells [72], and when assessing the effect of AgNPs on rainbow trout hepatocytes, ROS production was not detected and cytotoxicity was mainly associated with a decrease in mitochondrial activity and membrane integrity. The toxicity of AgNPs associated with membrane disruption has been reported in many studies. The formation of pits and pores in the cell membrane of fungus *C. albicans* was observed, and the authors suggested that AgNPs can attack the lipid bilayers of the cell membrane and affect its permeability, leading to ion leakage, pore formation, and cell death [73].

### 3.2. Interaction of AgNPs with Cells

**Figure 6:**
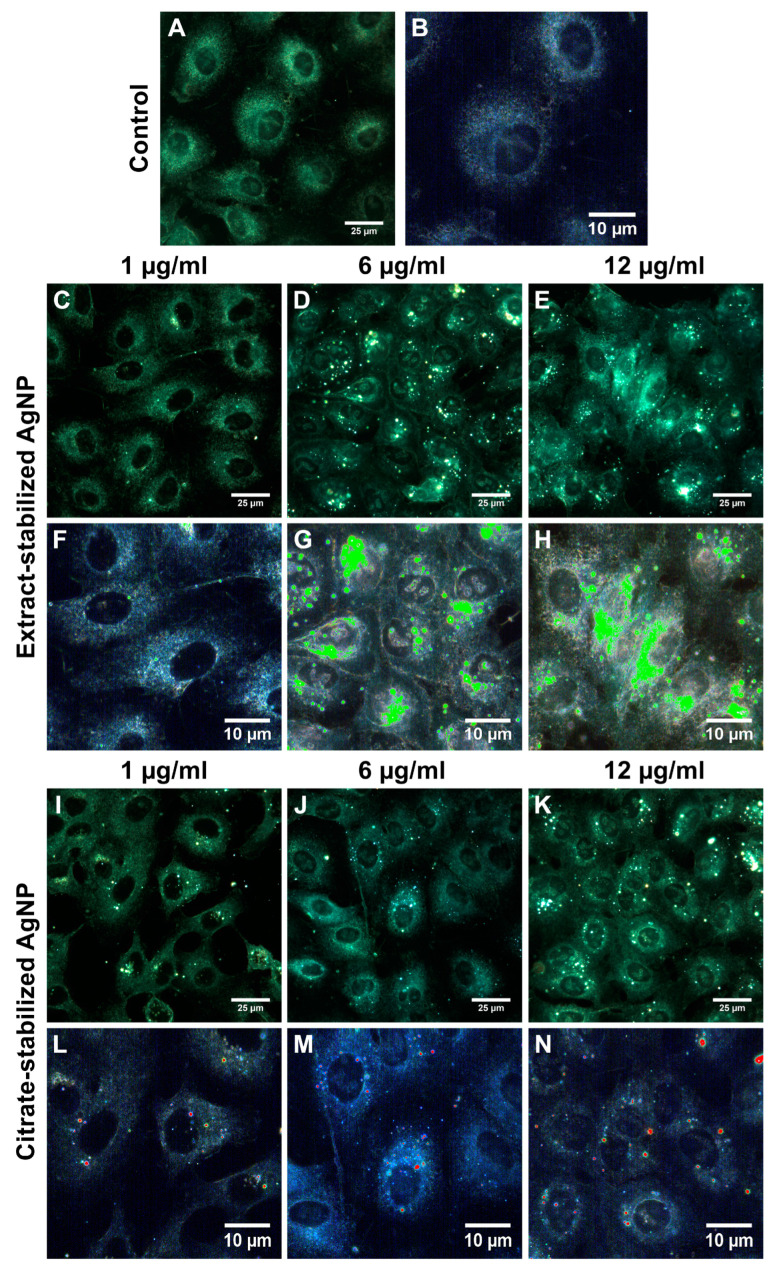
Dark-field images (**A**,**C**–**E**,**I**–**K**) and hyperspectral analysis (**B**,**F**–**H**,**L**–**N**) of A549 control cells (**A**,**B**) and A549 cells incubated with extract-(**C**–**H**) and citrate-(**I**–**N**) stabilized AgNPs at different concentrations (1 µg/mL (**C**,**F**,**I**,**L**), 6 µg/mL (**D**,**G**,**J**,**M**), and 12 µg/mL (**E**,**H**,**K**,**N**)) for 24 h.

**Figure 7:**
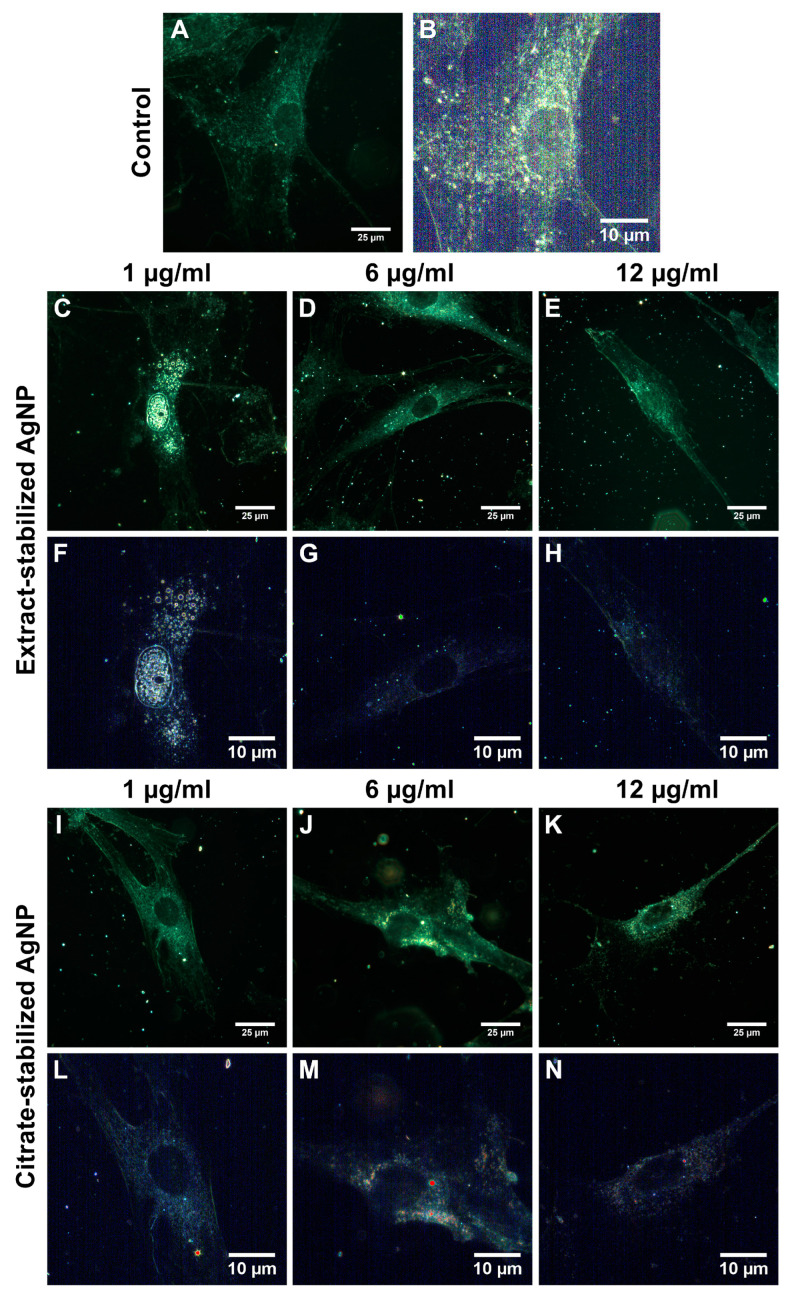
Dark-field images (**A**,**C**–**E**,**I**–**K**) and hyperspectral analysis (**B**,**F**–**H**,**L**–**N**) of iMSC control cells (**A**,**B**) and iMSC cells incubated with extract-(**C**–**H**) and citrate-(**I**–**N**) stabilized AgNPs at different concentrations (1 µg/mL (**C**,**F**,**I**,**L**), 6 µg/mL (**D**,**G**,**J**,**M**), and 12 µg/mL (**E**,**H**,**K**,**N**)) for 24 h.

To understand the mechanism of action of silver nanoparticles stabilized with citrate and the *Sphagnum* extract, the nanoparticles were visualized inside and on the cell surface using dark-field microscopy. After 24 h of co-incubation, the nanoparticles were mapped using hyperspectral imaging (Figure 6 and Figure 7). The hyperspectral dark-field imaging allows the detection of silver nanoparticles that penetrate into cells and cause morphological changes. The dark-field imaging and hyperspectral mapping revealed large quantities of extract-stabilized silver nanoparticles in A549 cells (Figure 6). 

The presence of single nanoparticles or small conglomerates was registered inside iMSC cells (Figure 7). The visualization of nanoparticle–cell interactions was recorded as video images (Supplementary Materials).

It is assumed that the internalization of negatively charged nanoparticles occurs by nonspecific binding to cationic sites of the plasma membrane and their subsequent endocytosis [74,75]. Chithrani and colleagues suggested that whey proteins bind to the surface of citrate-stabilized gold nanoparticles, forming a more stable gold nanoparticle–protein complex capable of interacting with cell surface receptors [74]. It is possible that the extract-stabilized silver nanoparticles are internalized by a similar process, which occurs when metabolically active compounds are taken up by cells. This process involves the invagination of a small portion of the plasma membrane, resulting in the formation of a membrane-bound vesicle that contains the nanoparticles. Although we did not observe a significant internalization of citrate-stabilized nanoparticles, there are suggestions that AgNP uptake may increase after aggregation [76]. Chithrani and Chan reported that 14 nm nanoparticles coated with transferrin were absorbed only when combined into groups consisting of at least six particles [77]. 

Scanning electron microscopy and EDX were additionally used to evaluate the binding of nanoparticles to the cell membrane (Figure S3, Supplementary Materials). The mapping showed that there are no silver nanoparticles on cell membranes, which is consistent with the previously published data. No silver nanoparticles on the membrane surface were found using TEM in [56], and the nanomaterials were only observed inside cells.

### 3.3. Spheroids

A number of studies have shown that the use of plant extracts makes it possible to obtain silver nanoparticles with anticancer activity [78,79,80]. The data collected during this study showed that silver nanoparticles synthesized in the presence of the sphagnum extract have a more pronounced toxic effect on iMSC cells compared to the previously reported data.

**Figure 8:**
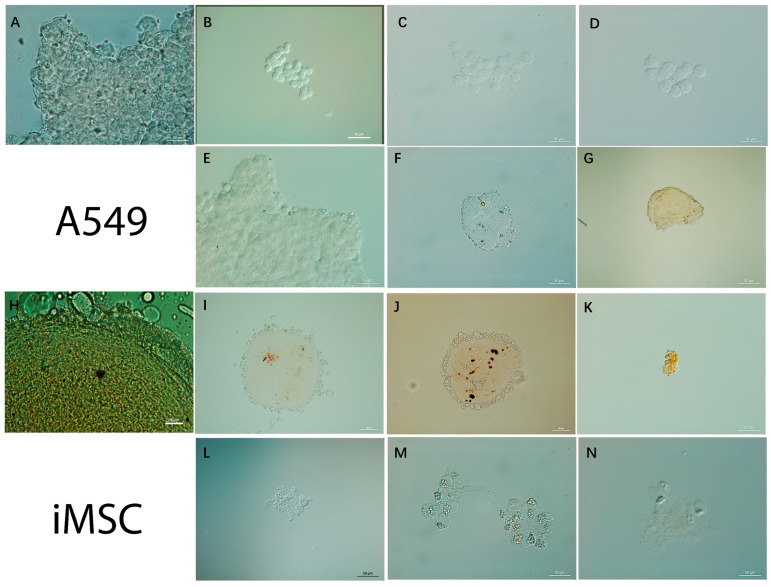
Visualization of the formation of multicellular spheroids from cells treated with silver nanoparticles using optical microscopy: control (**A**,**H**); spheroids from A549 cells incubated with extract-stabilized silver nanoparticles at concentrations of 1, 6, and 12 μg/mL (**B**, **C**, and **D**, respectively); spheroids from A549 cells incubated with citrate-stabilized silver nanoparticles at concentrations of 1, 6, and 12 μg/mL (**E**, **F**, and **G**, respectively); spheroids from iMSC cells incubated with extract-stabilized silver nanoparticles at concentrations of 1, 6, and 12 μg/mL (**I**, **J**, and **K**, respectively); spheroids from iMSC cells incubated with citrate-stabilized silver nanoparticles at concentrations of 1, 6, and 12 μg/mL (**L**, **M**, and **N**, respectively).

It has been reported that spheroid cultures exhibit in vivo-like characteristics similar to those of tissue cultures and are the best adapted model for assessing the characteristics of cell resistance in vitro [81]. Spheroids and 2D cultures have different degrees of sensitivity, with the monolayer being more sensitive and the spheroids more similar to tissues in vivo. Therefore, evaluating the viability of cells under the treatment with nanoparticles using more complex models, such as spheroids, in addition to monolayer cultures, is relevant to obtain results close to real systems [82,83]. In the present study, the formation of multicellular spheroids from cells treated with silver nanoparticles stabilized with the sphagnum extract or citrate was evaluated using optical light microscopy. The formation of 3D structures was shown in all the studied variants (Figure 8). 

**Figure 9:**
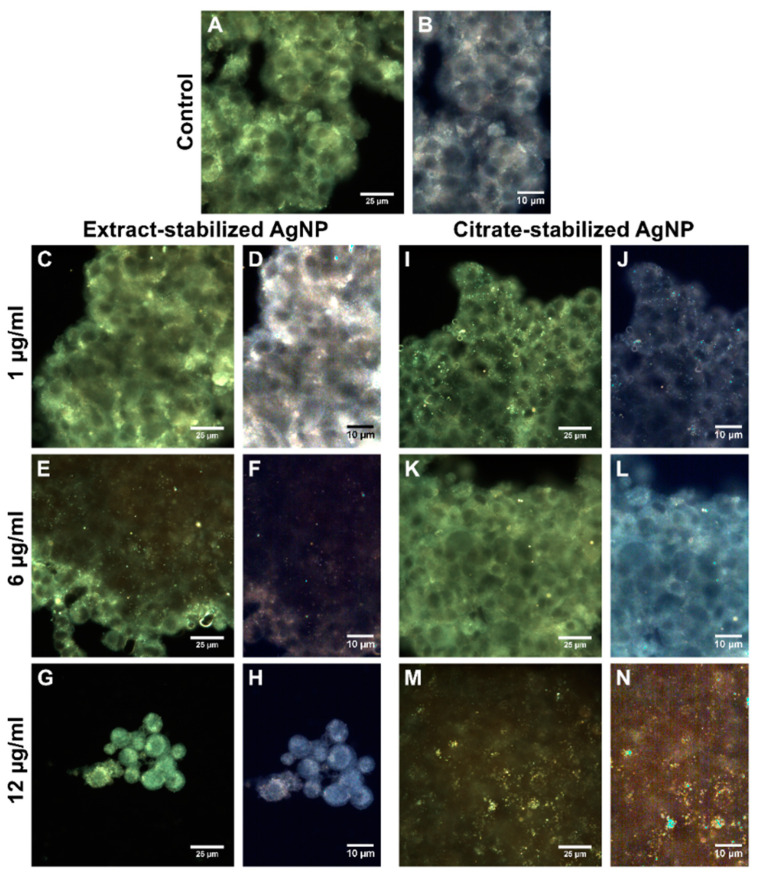
Visualization of spheroids from A549 control cells (**A**,**B**), after incubation with extract-stabilized (**C**–**H**) and citrate-stabilized (**I**–**N**) silver nanoparticles for 24 h.

**Figure 10:**
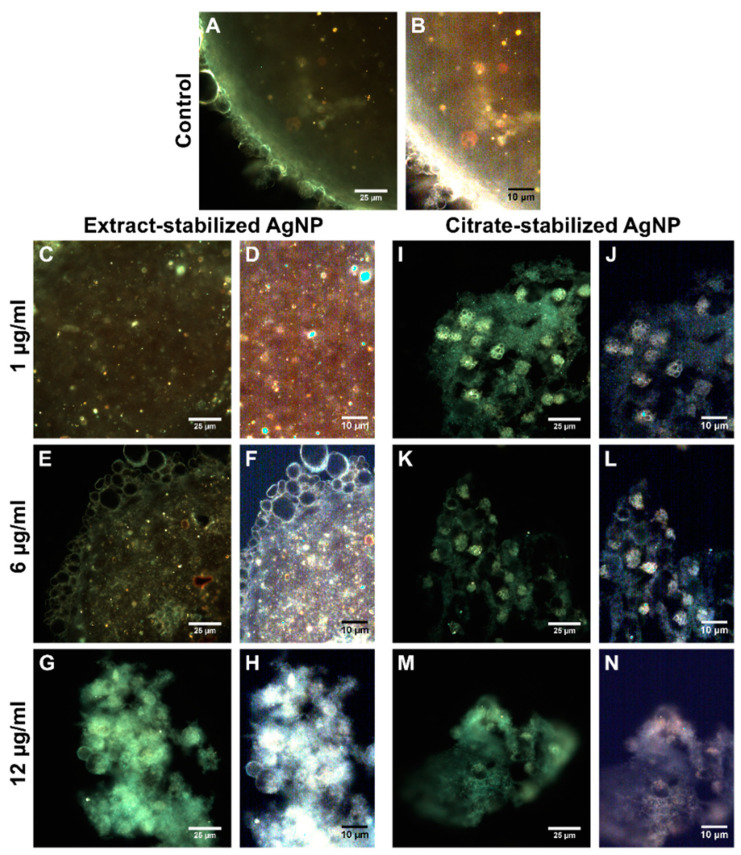
Visualization of spheroids from iMSC control cells (**A**,**B**) and iMSC cells incubated with extract-stabilized (**C**–**H**) and citrate-stabilized (**I**–**N**) silver nanoparticles for 24 h.

Additionally, to evaluate the internalization of silver nanoparticles into cells in multicellular spheroids, a mapping of silver nanoparticles based on hyperspectral data was made (Figure 9 and Figure 10). 

It is known that, depending on the cell line used, spheroids may be looser or more compact, since some cell lines have greater cell–cell adhesion than others [84]. In the present study, it was found that extract-stabilized silver nanoparticles led to the formation of a looser spheroid structure in A549 cells. No such loose structures were observed previously when the cells were functionalized with magnetic nanoparticles or halloysite nanotubes [31]. Also, the looser spheroid structure was visualized when high concentrations of silver nanoparticles of both types were used. 

The use of hyperspectral microscopy made it possible to map nanoparticles both within the cells and in the intercellular space. In addition, as with the 2D monolayer, the internalization of citrate-stabilized nanoparticles was mapped to a greater extent in 3D spheroids. This is probably due to the fact that negatively charged nanoparticles can diffuse faster, which allows them to reach deep tissues [85]. In addition, it may be related to the size of nanomaterials [86]. The penetration profile may also vary depending on the size of the spheroid and the type of cell line [85]. 

## 4. Conclusions

The modification of the method for silver nanoparticle synthesis using environmentally friendly techniques based on the reduction and stabilization of AgNPs with the *Sphagnum* extract was carried out. The resulting nanoparticles were carefully characterized and compared to citrate-stabilized nanoparticles. Both types of nanoparticles showed characteristic peaks at 405–420 nm in the UV-vis absorbance spectra. The hydrodynamic diameter and zeta potential were 104 ± 1.9 nm and −34 ± 1.6, respectively, for extract-stabilized nanoparticles and 103.7 ± 1.6 nm and −40.6 ± 3.4, respectively, for citrate-stabilized nanoparticles. In the micrographs, the nanoparticles were of uniform spherical shape, had a size of 20–30 nm, and were well dispersed. The surface of the extract-stabilized nanoparticles was coated with metabolites from the moss extract, as evidenced by the results of FT-IR spectroscopy and elemental analysis. In general, the moss-derived nanoparticles were found to be more stable than the citrate-stabilized ones. A good sedimentation stability was observed in the pH range of 5–9 for the AgNPs stabilized with the moss extract and pH 5–7 for the AgNPs stabilized with citrate.

The decrease in cell viability, changes in cell morphology, and confluence were observed after incubation with both types of AgNPs. The extract-stabilized nanoparticles exhibited a stronger cytotoxic effect. Possible causes of such pronounced toxicity may be related to a specific interaction mechanism of nanoparticles with the cell. 

The nanoparticles synthesized using an extract of the moss at room temperature had similar properties to the nanoparticles synthesized at 90 °C, but the synthesis procedure used in the present study was greener as it did not include heating (and thus energy consumption).

## Figures and Tables

**Table 1:** Analysis of the cell confluence.

**Cellular Confluence, %**		**Control**	**Citrate**	***S. fallax***	**Citrate-Stabilized AgNPs**			**Extract-Stabilized AgNPs**		
					**1 µg/mL**	**6 µg/mL**	**12 µg/mL**	**1 µg/mL**	**6 µg/mL**	**12 µg/mL**
	A549	100	66 ± 6.1	100 ± 4.4	76 ± 6	63 ± 5.9	58 ± 4.6	49 ± 7.3	38 ± 4.5	25 ± 5.5
	iMSC	100	98 ± 5.3	77 ± 5.7	90 ± 5.3	63 ± 3.2	47 ± 3.5	68 ± 4.2	39 ± 3.1	16 ± 0.9

## Data Availability

Data are contained within the article and Supplementary Materials, and available from the corresponding author upon reasonable request.

## Supplementary Materials

The following supporting information can be downloaded at: https://www.mdpi.com/article/10.3390/biom14060611/s1, Figure S1: The change in the color of the solution during synthesis from pale yellow to yellowish brown; Figure S2: Characteristic extract and citrate of stabilized silver nanoparticles; Figure S3: EDX analysis of A549 cells, incubated for 24 h with extract-stabilized nanoparticles at concentration of 12 μg/mL; Figure S4: EDX analysis of A549 cells, incubated for 24 h with citrate-stabilized nanoparticles at concentration of 12 μg/mL.
