# Supplementary material for: Advances in the Toxicity Assessment of Silver Nanoparticles Derived from a Sphagnum fallax Extract for Monolayers and Spheroids
Source: Biomolecules. 2024 May 22;14(6):611. doi: 10.3390/biom14060611 (PMC11202274; doi:10.3390/biom14060611)
Supplement: Supplementary file 1 [file biomolecules-14-00611-s001.zip › biomolecules-2959148-supplementary.pdf]

Supplementary materials

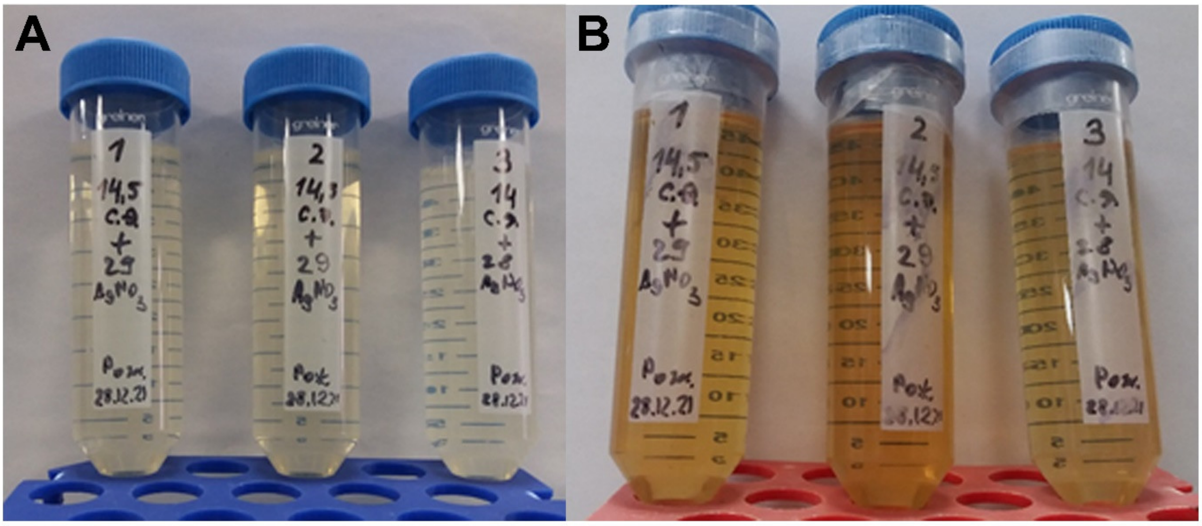

Figure S1. The change in the color of the solution during synthesis from pale yellow to yellowish brown. *S. fallax* moss extract together with $AgNO_3$ before synthesis (A); *S. fallax* moss extract together with $AgNO_3$ after synthesis (B).

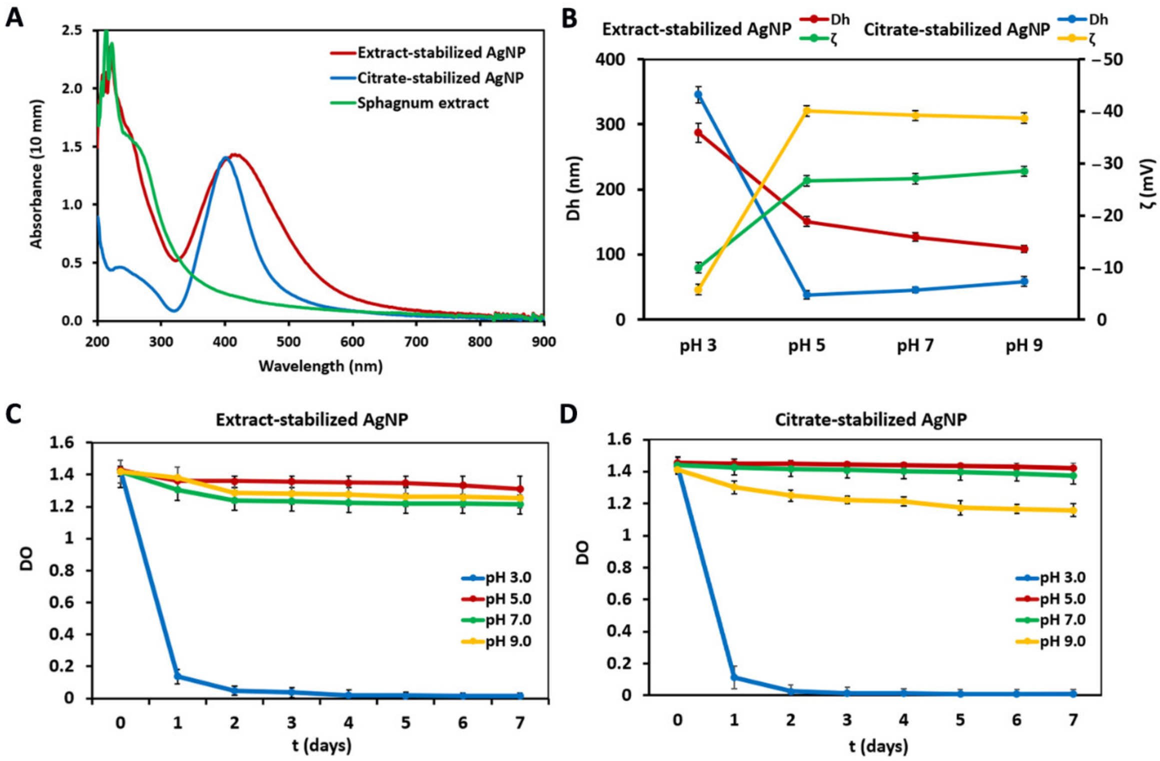

Figure S2. Characteristics of extract- and citrate-stabilized silver nanoparticles. Absorption spectra of AgNP (A); values of hydrodynamic diameter (Dh) and zeta potential (ζ) of AgNP at different pH values (B); AgNP suspension stability at different pH values assessed by dependence of optical density of AgNP suspensions on time (C, D).

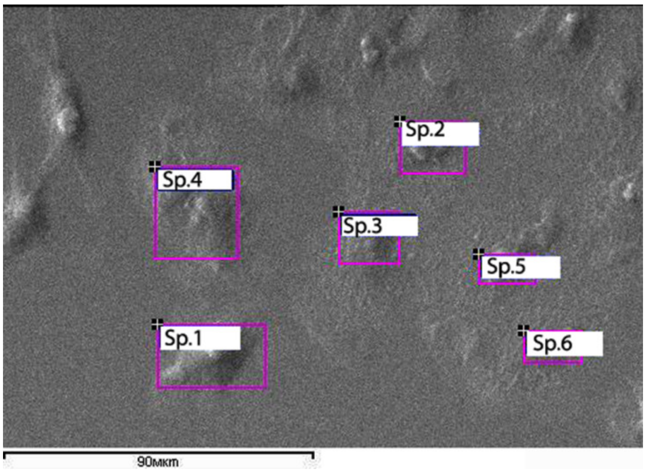

	O	Na	Al	Si	Cl	K	Ti	Zn	Sum
**Spectrum 1**	41.11	5.50	2.46	35.74	1.07	6.07	2.85	5.20	100.00
**Spectrum 2**	37.74	5.73	2.39	38.22	1.07	6.73	2.83	5.30	100.00
**Spectrum 3**	36.91	5.84	2.30	38.77	1.22	6.63	3.02	5.30	100.00
**Spectrum 4**	38.54	5.85	2.32	37.80	1.41	6.35	2.65	5.09	100.00
**Spectrum 5**	35.86	5.98	2.52	39.50	0.95	6.74	2.75	5.70	100.00
**Spectrum 6**	35.36	5.78	2.27	40.12	1.13	6.85	2.94	5.54	100.00
**Mean**	37.59	5.78	2.38	38.36	1.14	6.56	2.84	5.35	100.00
**St.Dev**	2.08	0.16	0.10	1.53	0.16	0.30	0.13	0.23	

Figure S3. EDX analysis of A549 cells, incubated for 24 h with extract-stabilized nanoparticles at a concentration of 12 μg/ml.

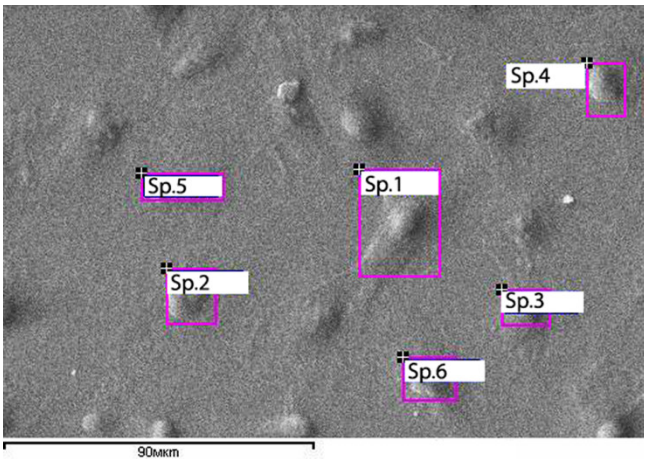

Spectrum	O	Na	Al	Si	K	Ti	Zn	Sum
**Spectrum 1**	43.32	5.37	2.35	35.45	5.91	2.48	5.13	100.00
**Spectrum 2**	40.33	4.97	2.43	38.38	6.39	2.85	4.66	100.00
**Spectrum 3**	41.08	4.93	2.42	37.69	6.42	2.67	4.79	100.00
**Spectrum 4**	42.55	5.25	2.40	36.37	5.90	2.56	4.98	100.00
**Spectrum 5**	40.35	5.45	2.50	37.75	6.33	2.58	5.04	100.00
**Spectrum 6**	39.86	5.17	2.36	38.56	6.43	2.94	4.67	100.00
**Mean**	41.25	5.19	2.41	37.37	6.23	2.68	4.88	100.00
**St.Dev**	1.39	0.21	0.05	1.22	0.25	0.18	0.20	

Figure S4. EDX analysis of A549 cells, incubated for 24 h with citrate-stabilized nanoparticles at a concentration of 12 μg/ml.
